# Structural-activity relationship of *Lycium barbarum* polysaccharides in immunomodulation: integrating molecular insights with target identification for therapeutic development

**DOI:** 10.3389/fimmu.2026.1730418

**Published:** 2026-01-26

**Authors:** Bo Wang, Jie Yang, Lijun Tao, Xuebing Zhou, Xiaoling Ding

**Affiliations:** People’s Hospital of Ningxia Hui Autonomous Region, Ningxia Medical University, Yinchuan, China

**Keywords:** immunomodulatory, *Lycium barbarum*, polysaccharides, structure-activity relationship, therapeutic development

## Abstract

The immunomodulatory potential of *Lycium barbarum* polysaccharides (LBP) is well-established, yet the intricate structure-activity relationships (SAR) underlying these effects require clarification to advance therapeutic applications. This review synthesizes current knowledge on how specific structural parameters of LBP, including molecular weight, monosaccharide composition, glycosidic linkage types, and chemical modifications influence its immunoregulatory functions. Key findings reveal a non-linear dependence of LBP’s immunomodulatory activity on molecular weight. Fractions within the medium molecular weight range (10^5^–10^6^ Da) often demonstrate optimal efficacy, which is attributed to their capacity for facilitating multivalent binding to pattern recognition receptors (PRRs). Furthermore, a high content of arabinose and galactose is a critical structural determinant, with arabinogalactan-like motifs serving as key recognition elements for immune cell activation. Mechanistically, LBP orchestrates immune responses through multi-target pathways. It directly modulates macrophage polarization via the STAT1/STAT6 pathways, promotes dendritic cell maturation through NF-κB and Notch signaling, and influences T-cell differentiation. Concurrently, LBP exerts indirect immunomodulatory effects via the gut microbiota-immune axis by enriching beneficial bacteria and their immunoregulatory metabolites, such as short-chain fatty acids. Despite robust preclinical evidence, clinical translation is hampered by the heterogeneity of LBP preparations. This review underscores the necessity of standardizing LBP based on SAR insights to develop precision immunomodulators for therapeutic applications.

## 1 Introduction

The immune system, a sophisticated defense network, requires precise regulation to maintain homeostasis and combat diseases. Dysregulation of immune responses is a hallmark of various pathological conditions, ranging from immunodeficiency leading to increased susceptibility to infections and cancer, to hyperactivation causing autoimmune disorders and chronic inflammation (1–3). Consequently, the development of safe and effective therapeutic agents capable of modulating immune function remains a central goal in biomedical research. Among various candidates, bioactive polysaccharides derived from natural sources have garnered significant attention due to their broad immunomodulatory activities and favorable safety profiles (4, 5). These polysaccharides, particularly those extracted from traditional Chinese herbs, act as agonists for pattern recognition receptors, enabling them to interact with innate immune cells such as macrophages and dendritic cells, thereby influencing adaptive immunity and showcasing considerable potential as ideal immunomodulators or adjuvants (6–9). However, a major obstacle to the clinical application and mechanistic elucidation of natural polysaccharides is the frequently ambiguous relationship between their structure and biological activity. Natural polysaccharides are typically heterogeneous, high-molecular-weight polymers exhibiting significant variations in critical parameters such as molecular weight, monosaccharide composition, glycosidic linkage types, and branching patterns (10, 11). This inherent complexity poses considerable challenges in isolating homogeneous fractions, delineating precise structure-activity relationships (SAR), and identifying the key active determinants responsible for their immunomodulatory effects. Therefore, systematically deciphering how the fine chemical structures of polysaccharides dictate their specific immunological functions has emerged as a crucial, yet unresolved, scientific question in the field. Addressing this issue is imperative not only for fundamental scientific exploration but also as a prerequisite for translating natural polysaccharides into standardized, potent, and controllable immunotherapeutic agents.

*Lycium barbarum* L., a perennial deciduous shrub of the Solanaceae family, has been used in traditional Chinese medicine for over two thousand years, with its dried ripe fruits known as goji berries (12). In modern research, *Lycium barbarum* polysaccharides (LBP) are recognized as one of the primary bioactive components, serving as a key material basis for various pharmacological effects including immune regulation, antioxidation, antitumor activity, and neuroprotection (13). Among immunomodulatory natural polysaccharides, LBP stands out as one of the most extensively studied representatives (14). Accumulating preclinical evidence demonstrates that LBP possesses multifaceted immunoregulatory functions (15, 16). These include activating innate immune cells, modulating adaptive immunity, and exerting systemic immune effects indirectly via the gut microbiota-immune axis. These activities suggest substantial potential for LBP in immunotherapeutic interventions for infections, cancer, and autoimmune diseases. Despite substantial evidence supporting LBP’s immunomodulatory potential, significant knowledge gaps persist. Firstly, no consensus exists regarding the critical structural features dictating LBP’s immune activity, such as the most efficacious molecular weight range, essential monosaccharide residues, crucial glycosidic linkages, or specific functional groups, with findings sometimes being contradictory across studies. Secondly, while the immunomodulatory mechanisms of LBP have been partially uncovered, a comprehensive understanding of how it precisely regulates immune cells through a multi-target and multi-pathway network remains incomplete. Particularly striking is the “evidence vacuum” in translating LBP from preclinical research to clinical application, characterized by a scarcity of well-designed human randomized controlled trials with immune parameters as primary endpoints. This disconnect between basic research and clinical translation is largely attributable to the non-standardized nature of LBP preparations. Thus, systematically correlating the chemical structure of LBP with its immunological effects and subsequently developing standardized, high-potency LBP formulations based on this knowledge is critical for realizing its therapeutic promise.

This review aims to systematically consolidate current advances in understanding the structure-immunoactivity relationship of LBP and their molecular mechanisms, while critically assessing the status of clinical translation. It will integrate high-resolution structural descriptors, including molecular weight (MW), monosaccharide composition, glycosidic linkage motifs, and chain conformation with molecular immunology insights, such as pattern recognition receptor (PRR) engagement, intracellular signaling pathways, and microbiota-immune crosstalk, to establish a translational roadmap for standardizing and developing LBP-based immunomodulators. The review will further elaborate on the molecular basis of LBP’s immunoregulatory functions, encompassing its modulation of immune cell activities, associated signal transduction mechanisms, and interactions along the gut microbiota-immune axis. Finally, it addresses current limitations in clinical research and proposes future directions, emphasizing high-resolution structural characterization, synthetic biology approaches, and targeted immunotherapy strategies, thereby providing a theoretical framework for developing precision LBP immunomodulators both theoretically and practically.

## 2 The structural characteristics of Lycium polysaccharides

LBP are macromolecules composed of various monosaccharide units linked by glycosidic bonds. The unique bioactivities of LBP are largely determined by their intricate structural features, including molecular weight, monosaccharide composition and molar ratios, types and sequences of glycosidic linkages, as well as chain conformation and branching patterns. To systematically characterize these complex structural parameters, a combination of advanced analytical techniques is employed. For instance, molecular weight and its distribution are typically analyzed using high-performance gel permeation chromatography (HPGPC). Monosaccharide composition can be determined by gas chromatography (GC) or high-performance liquid chromatography (HPLC), while detailed structural information on glycosidic linkage types and backbone conformation is often elucidated through nuclear magnetic resonance (NMR) spectroscopy, mass spectrometry (MS), and Fourier transform infrared (FT-IR) spectroscopy. Collectively, the application of these techniques has significantly advanced our understanding of LBP’s chemical structure. Key research findings on the structural characteristics of LBP are summarized in **Table 1** for a comprehensive overview.

**Table 1:** molecular weight, monosaccharide composition, and structural characteristics of LBP.

Name	Extraction & purification	Source	Molecular weight (kDa)	Monosaccharide composition (molar ratio)	Key structural features	Structural characterization method	Biological activities	Ref
LBP-a4	HWE, ultrafiltration membranes with MWCO	Lycium barbarum	10.2	Fuc: Ara: Xyl: Glu: Man: Gal=19.6:17.1:8.2:10.7:15.1:46.9	ND	GC, HPLC, AFM	Anti-cancer activity	(17)
LBP-p8	HWE, ultrafiltration membranes with MWCO	Lycium barbarum	6.50 × 10^3^	Fuc: Rha: Ara: Xyl: Glu: Man: Gal=5.7:2.5:21.5:8.4:4.6:23.3:33.9	ND	GC, HPLC, AFM	Promote tumor activity	(17)
PLBP-I-I	HWE, DEAE Sepharose Fast Flow, Sepharose 6FF	Lycium barbarum	599.5	Ara: Rha: Xyl: Gal: GalA=25.7:12.4:0.5:27.5:33.9:	typical pectic polysaccharides, both AG-I and AG-II	GC, SEC, FT-IR, NMR	Antioxidant activity	(18)
PLBP-II-I	HWE, DEAE Sepharose Fast Flow, Sepharose 6FF	Lycium barbarum	716.6	Ara: Rha: Xyl: Gal: GalA=26.6:20.8:1.9:7.6:43.1	HG region (1→4-linked GalA) with methyl esterification	GC, SEC, FT-IR, NMR	Antioxidant activity	(18)
LBPs-1	HWE, ultrafiltration membrane separation	Lycium barbarum	1.912	Rha: Ara: Gal: Glu: Xyl: Man: GluA=0.10:0.58:0.31:97.39:0.25:0.72:0.64	–	HPSEC-RID-MALLS, HPAEC-PAD, Congo red experiment, FT-IR, NMR, AFM	Antioxidant activity	(19)
LBPs-2	HWE, ultrafiltration membrane separation	Lycium barbarum	7.481	Fuc: Rha: Ara: Gal: Glu: Xyl: Man: GluA= 0.38:0.82:7.58:3.93:80.56:2.49:3.64:0.60	→4)-β-Galp-(1→, α-Glcp-(1→, →3)- α-Glcp-(1→, β-Glcp-(1→, →3,4)-β-Arap-(1→, →3)-α-Arap-(1→ and →4)-α-D-GlcpA-(1→	HPSEC-RID-MALLS, HPAEC-PAD, Congo red experiment, FT-IR, NMR, AFM	Antioxidant activity, immunomodulatory activity	(19)
LBPs-3	HWE, ultrafiltration membrane separation	Lycium barbarum	46.239	Fuc: Rha: Ara: Gal: Glu: Xyl: Man: GluA= 0.50:2.03:49.27:28.35:13.38:2.42:2.73:1.32	→3,4)-α-Arap-(1→, →3,4)-α-Galp-(1→, →3)-α-Galp-(1→, →4)-β-Arap-(1→, β-Arap-(1→, →3,4)-α-Galp-(1→ and →3)-α-GlcpA-(1→	HPSEC-RID-MALLS, HPAEC-PAD, Congo red experiment, FT-IR, NMR, AFM	Antioxidant activity, immunomodulatory activity	(19)
XLBP-I-I	HWE, anion exchange chromatography and gel filtration	Lycium barbarum	419.6	Ara: Rha: Xyl: Gal: GluA: GalA=26.5:12.9:0.7:16.8:2.3:40.8	HG consisting of α-(1→4)-linked GalA residues with RG-I regions	GC, SEC, FT-IR, NMR	Protects against intestinal endoplasmic reticulum stress	(20)
LBP 2-1	HWE, DEAE-cellulose 52 column, Sephadex G-75 column	Lycium barbarum	102.2	Man: Rha : GalA: Glc : Gal: Ara=0.97:6.20:24.67:3.90:20.42:43.84	→2)-α-L-Rhap-(1 → 4)-α-DGalAp-(1 → 6)-β-D-Galp-(1 →	FT-IR, HPSEC-RID-MALLS, HPLC, NMR	Anti-ulcerative colitis	(21)
LBP3b	HWE, DEAE cellulose column and Sephadex G-150	Lycium barbarum	4.92	Man: Rha: Glu: Gal: Xyl= 5.52:5.11:28.06:1.00:1.70	Both α- and β-glycosidic linkages in pyranose ring	HPLC, HPGPC, UV, FT-IR, NMR and SEM	Hypoglycemic activity	(22)
LBP-s-1	HWE, macropolorus resin S-8, DEAE column	LyciuCrum	1.92 × 10^3^	Rha: Ara: Xyl: Man: Glu: Gal: GalA = 1.00:8.34:1.25:1.26:1.91:7.05:15.28	Both α- and β-glycosidic linkages	HPSEC, GC, HPLC, FT-IR, NMR	Hypoglycemic effect	(23)
W_H_	HWE,	Lycium barbarum	49.44	Man: Rib: Rha: GlcA: GalA: Glc: Gal: Ara: Fuc=2.79:0.57:4.17:1.64:19.75:4.59:20.74:39.13:6.62	ND	HPLC, HPSEC-MALLS-RI, FT-IR, NMR, AFM	ND	(24)
A_H_	High-temperature acid extraction	Lycium barbarum	199.2	Man: Rib: Rha: GlcA: GalA: Glc: Gal: Ara: Fuc=3.78:1.00:9.88:3.84:23.67:3.44:24.11:24.31:5.37	homogalacturonan (HG) regions	HPLC, HPSEC-MALLS-RI, FT-IR, NMR, AFM	ND	(24)
AL_H_	High-temperature alkali extraction	Lycium barbarum	223.1	Man: Rib: Rha: GlcA: GalA: Glc: Gal: Ara: Fuc=7.39:0.70:8.21:2.60:6.34:4.14:25.23:29.17:16.22	–	HPLC, HPSEC-MALLS-RI, FT-IR, NMR, AFM	ND	(24)
A_L_	Low-temperature acid extraction	Lycium barbarum	2334	Man: Rha: GlcA: GalA: Glc: Gal: Ara: Fuc=2.24:7.47:1.84:28.24:9.06:20.54:22.94:7.68	ND	HPLC, HPSEC-MALLS-RI, FT-IR, NMR, AFM	ND	9
AL_L_	Low-temperature alkali extraction	Lycium barbarum	7162	Man: Rha: GlcA: GalA: Glc: Gal: Ara: Fuc=4.11:7.66:1.61:14.53:6.18:22.23:37.26:6.43	branched rhamnogalacturonan I (RG-I) domains	HPLC, HPSEC-MALLS-RI, FT-IR, NMR, AFM	ND	(24)
W_H_AL_H_	Water extraction residue alkali extraction	Lycium barbarum	207.1	Rha: GalA: Glc: Gal: Ara: Fuc=16.27:13.30:2.65:25.65:37.15:4.98	ND	HPLC, HPSEC-MALLS-RI, FT-IR, NMR, AFM	ND	(24)
W_H_AL_L_	Low temperature alkaline leaching enhances warm water extraction	Lycium barbarum	6371	Man: Rha: GlcA: GalA: Glc: Gal: Ara: Fuc=2.67:11.99:0.88:22.08:4.19:12.81:43.89:1.52	ND	HPLC, HPSEC-MALLS-RI, FT-IR, NMR, AFM	ND	(24)
A_H_ AL_H_	Alkali extraction of residue after acid leaching	Lycium barbarum	194.6	Man: Rib: Rha: GlcA: GalA: Glc: Gal: Ara: Fuc=6.98:1.02:13.9:4.01:25.46:3.38:22.25:17.73:5.27	ND	HPLC, HPSEC-MALLS-RI, FT-IR, NMR, AFM	ND	(24)
A_L_AL_L_	Alkali extraction	Lycium barbarum	2294	Man: Rha: GlcA: GalA: Glc: Gal: Ara: Fuc=2.06:11.04:1.23:31.06:5.33:13.44:31.84:4.00	ND	HPLC, HPSEC-MALLS-RI, FT-IR, NMR, AFM	ND	(24)
LBPS02	HWE, DEAE-52 column and a Sepharose G-100 column	Lycium barbarum	68	Rha: Glu:	containing 1→3, 1→4 and 1→6 linkages	HPLC, UV, HPLC,	Neurodegenerative disorders	(25)
p-LBP	HWE, Sepharcyl S400 gel column	Lycium barbarum	64	Fuc: Rha: Ara: Gal: Glc: Xyl: GalA: GluA= 1.00: 6.44: 54.84: 22.98: 4.05: 2.95: 136.98: 3.35	→4-α-GalpA-(1→ units, interspersed with →2-α-Rhap-(1→ residues to form RG-I regions.	HPAEC-PAD, HPLC, HPSEC, FT-IR, GC–MS, and NMR	ND	(26)
LBP-3	HWE, DEAE-Crystarose Fast Flow column	Lycium barbarum	67.4	Ara: Gal= 1.00:1.56	complex arabinogalactan with a 1,3-β-galactan backbone and multi-branched side chains rich in arabinose and galactose	HPLC, FT-IR, GC-MS, NMR	Alzheimer’s disease	(27)
LAP	HWE,	Lycium arabicum	ND	Rha: Ara: Gal: Glu: Man= 4.7:1.5:1:8.7:16.4:5.6	glucose-dominated backbone with 1→4 and/or 1→6 linkages	GC-MS, FT-IR, NMR, SEM, XRD	Anti-oxidant activities	(28)
LBPA	HWE, DEAE Sepharose Fast Flow column, Sephacryl S-200HR column	Lycium barbarium	470	Ara: Gal: GlcpA: Rha= 9.2:6.6:1.0:0.9	complex arabinogalactan with a β-D-(1→6)-galactan backbone and multi-branched side chains rich in arabinose, rhamnose, and glucuronic acid	HPGPC, GC, GC-MS, NMR	ND	(29)
LBP-W	HWE, DEAE Fast Flow column	Lycium barbarum	112.97	Ara: Gal: Rha= 55.6: 35.5: 8.0	→6)-β-Galp (1 → residues with branches composed of α-Araf, β-Galp and α-Rhap residues	HPGPC, FT-IR, HPLC, GC-MS, NMR	Anti-obesity effects	(30)
LRWP-Ap	HWE, DEAE-Cellulose, DEAE-Sepharose Fast Flow	Lycium ruthenicum Murray	4.34	GalA: Ara: Gala: 75.81: 15.41: 8.78	pectic polysaccharide with a →4)-α-D-GalpA-(1→ backbone	HPLC, HPGPC, NMR	Anti-inflammatory	(31)
LRGP 1	HWE, DEAE-Cellulose-52 anion-exchange column, Sephadex G-100 column	Lycium ruthenicum Murr	56.2	Rha: Ara: Xyl: Man: Glu: Gal= 0.65: 10.71:0.33:0.67:1:10.41	backbone of →3)-Gal-(1→ linkages, and	HPGPC, GC, GC-MS, ESI-MS	ND	(32)
LRP4-A	HWE, DEAE-cellulose column	Lycium ruthenicum Murr	105	Rha: Ara: Glu: Gala=1:7.6:0.5:8.6	→6)-β-D-Galp-(1→ residues	HPGPC, GC, IR, NMR, and ESI-MS	ND	(33)
LRP-S2A	HWE, DEAE column, Sephacryl S-500 column	Lycium ruthenicum Murr	2.65 x 10^3^	Rha: Ara: Gal: Glc: GlcA=1.00: 2.07: 0.57: 2.59: 4.33	comprises 6-O-Me-α-(1→4)-GlcpA, 2-O-acetyl-α-(1→4)-Glcp, α-(1→2,4)-Rhap, β-(1→3)-Galp, and α-(1→3,5)-Araf	HPGPC, GC, FT-IR, NMR,	Osteoporosis	(34)
LBLP5-A	HWE, DEAE-52column, Sephadex G-100 column	lycium barbarum leaves	113.3	Rha: Ara: Gal= 0.5:1.9:1.0	Linear chain of →3)-β-D-Galp-(1→ residues	HPGPC, GC, FT-IR, ESI–MS, GC–MS, NMR	Anti-oxidativity	(35)

Arabinose (Ara), rhamnose (Rha), xylose (Xyl), galactose (Gal) and Galacturonic acid (GalA), ND, not detected.

### 2.1 Molecular weight

Molecular weight (MW), one of the most critical physicochemical parameters of polysaccharides, directly influences their solubility, spatial conformation, receptor-binding capacity, and *in vivo* metabolic fate (36–38). The determination of LBP molecular weight primarily relies on chromatographic techniques coupled with various detectors. Commonly used methods include gel permeation chromatography (GPC), high-performance gel permeation chromatography (HPGPC), and size exclusion chromatography (SEC), often in combination with differential refractive index detectors and multi-angle laser light scattering (MALLS) (39). GPC, based on the separation principle of molecular size, is a straightforward technique where larger molecules are eluted first (40). By employing a calibration curve constructed with standards of known molecular weight, the relative molecular weight of the polysaccharide can be estimated, providing basic information on its molecular weight distribution. In comparison, HPGPC offers higher separation efficiency, faster analysis speed, and better reproducibility, enabling more precise determination of molecular weight parameters such as the weight-average Mw, number-average molecular weight (Mn), and polydispersity index (PDI) (41). For instance, Wei et al. (42) utilized HPGPC to analyze the molecular weights of black goji berry polysaccharide fractions EFP-0, EFP-1, EFP-2, EFP-3, and EFP-4, which ranged from 4.4 × 10^5^ to 7.8 × 10^6^ Da. To obtain absolute molecular weight without relying on standards, the coupling of SEC with MALLS and a refractive index detector (HPSEC-MALLS-RID) has become the gold standard (24). This technique directly determines the absolute weight-average molecular weight (Mw) and the root-mean-square radius of gyration (Rz), significantly enhancing the depth and accuracy of LBP molecular weight characterization. It is important to note that LBP is not a single-molecular-weight substance but a complex mixture of polysaccharide chains with varying degrees of polymerization. Research data indicate that the molecular weight of LBPs can span a wide range, from tens to thousands of kDa (13). These substantial variations are primarily attributed to differences in the raw material source, extraction methods, and purification processes, as summarized in **Table 1**.

### 2.2 Monosaccharide composition

The monosaccharide composition is a fundamental determinant of the bioactivity of LBP. Analysis of monosaccharide composition primarily relies on chromatographic and spectroscopic techniques, such as high-performance liquid chromatography (HPLC), ion chromatography (IC), gas chromatography-mass spectrometry (GC-MS), and Fourier transform infrared spectroscopy (FT-IR) (43, 44). Notably, high-pH anion-exchange chromatography with pulsed amperometric detection (HPAEC-PAD) allows for the direct and highly sensitive detection of hydrolyzed monosaccharides without derivatization, simplifying the analytical procedure while ensuring accurate results (45). Furthermore, the application of advanced techniques like UHPLC-QTRAP-MS/MS has recently elevated the sensitivity and specificity of monosaccharide analysis to new heights, offering rapid analysis, molecular weight information, and strong anti-interference capabilities suitable for the precise quantification of trace monosaccharides in complex matrices (46). Several studies have characterized the monosaccharide profiles of various LBP fractions using these methods. For instance, Qin et al. (47) determined via HPLC that LRPS is primarily composed of arabinose (41.2%), galactose (33.6%), and glucose (10.8%). Similarly, Peng et al. (48), using IC and FT-IR, reported that LBPT consists of Rha, Ara, Gal, Glu, Xyl, and Man in a molar ratio of 1.00: 11.35: 6.10: 0.56: 1.08: 0.71, with glycosidic linkages mainly in the α-D-arabinose and β-D-galactopyranose configurations.

In summary, LBP is a heteropolysaccharide composed of various monosaccharides, including arabinose, galactose, glucose, rhamnose, mannose, and xylose, linked by glycosidic bonds. The presence of galacturonic acid also indicates the existence of acidic polysaccharide components within LBP. It is crucial to emphasize that the specific monosaccharide composition and molar ratios vary significantly depending on the *Lycium barbarum* variety, extraction method, purification process, and analytical technique used, as summarized in **Table 1**.

### 2.3 Chemical structure

LBP are not a single chemical entity but represent a class of structurally complex and highly heterogeneous acidic heteropolysaccharides. They often form covalent complexes with proteins as glycoproteins or proteoglycans, exhibiting significant diversity in their chemical composition and structural frameworks (49). The elucidation of LBP’s precise chemical structure relies on an integrated approach combining various chemical methods and modern instrumental techniques. These techniques include infrared spectroscopy, periodate oxidation, Smith degradation, methylation analysis, liquid chromatography, and one-/two-dimensional nuclear magnetic resonance spectroscopy (26, 50). Despite the technical challenges inherent in polysaccharide structural analysis, significant progress has been made in characterizing specific LBP components. For example, Zeng et al. (45) employed methylation analysis and NMR to characterize two polysaccharide fractions, LBPs-2 and LBPs-3. Their analysis identified key glycosidic linkages in LBPs-2, such as →4)-β-Galp-(1→ and →4)-α-D-GluAp-(1→. LBPs-3 was characterized by linkages including →3,4)-α-Arap-(1→ and →3)-α-Galp-(1→. In another study focusing on a water-soluble arabinogalactan protein (LRGP3) from *Lycium ruthenicum*, researchers determined its protein content to be 1.7%, rich in hydroxyproline. By comprehensively utilizing techniques such as partial acid hydrolysis, methylation analysis, ESI-MS, and NMR, the main chain of β-D-galactopyranose residue was identified as (1→3) - linked and replaced by galactose or arabinose groups at the *O*-6 position, forming a highly branched structure (51).

**Figure 1:**
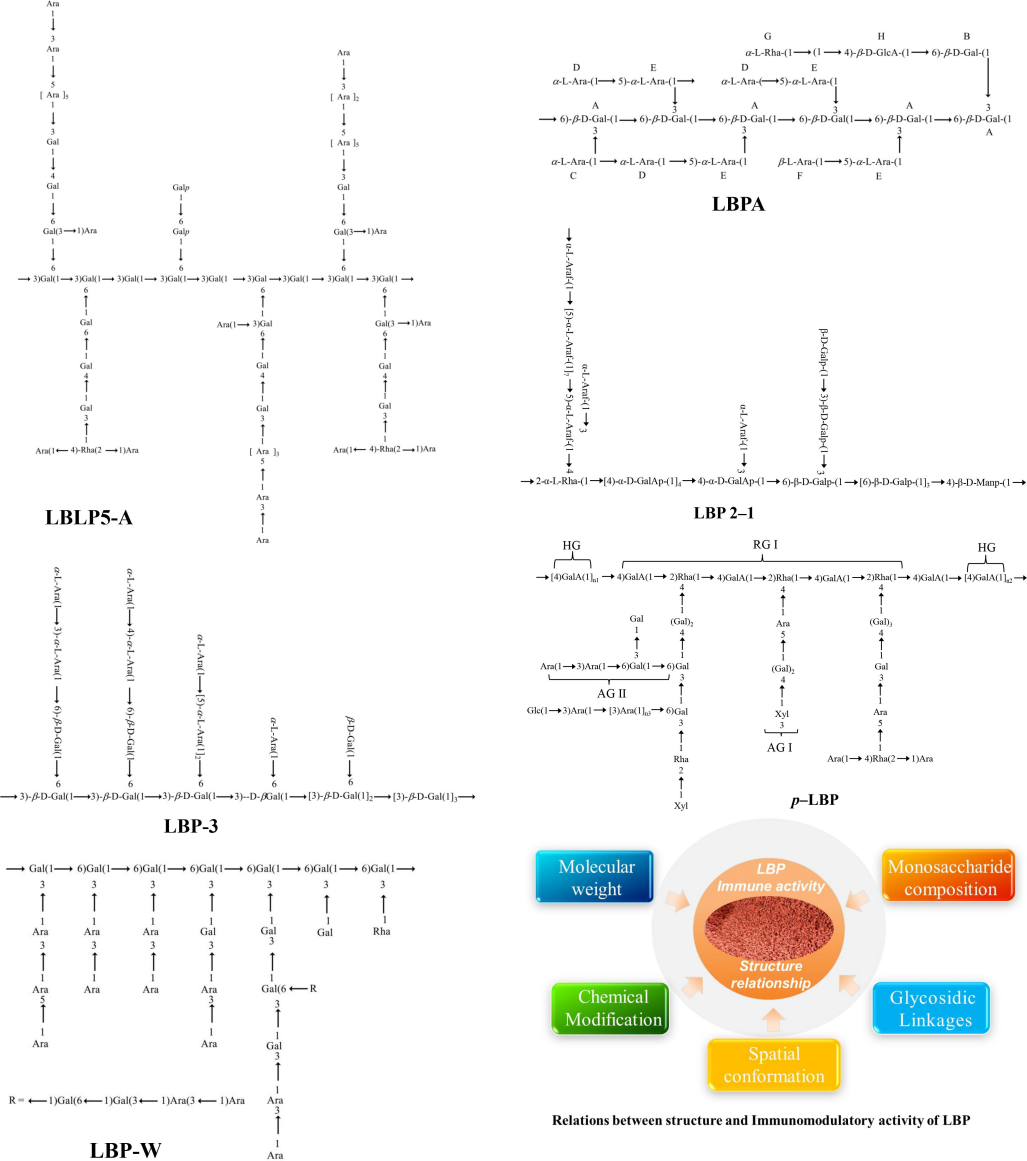
Representative structural features of polysaccharides from *Lycium barbarum* and their conformational relationships.

It is important to emphasize that the chemical structure of LBP varies considerably depending on the plant source and preparation methodologies. For a clear overview, existing literature on the chemical structures of LBP and its derivatives is summarized in **Table 1**, and the structural features of LBP have been shown in **Figure 1**.

## 3 Factors affecting the structure of *Lycium barbarum* polysaccharides

### 3.1 The effect of extraction methods on the structure of *Lycium barbarum* polysaccharides

A growing body of evidence indicates that the choice of extraction method not only affects the yield of polysaccharides but also profoundly alters their fine chemical structure, leading to significant differences in their bioactivity. Among various techniques, hot water extraction (HWE) is the most classical and widely used method for LBP extraction (52). It utilizes high-temperature water to disrupt cell walls and enhance polysaccharide solubility. However, HWE is a double-edged sword: while it effectively extracts water-soluble polysaccharides, prolonged heating (typically 80-100 °C) can induce random hydrolysis of glycosidic bonds, resulting in decreased molecular weight and potential degradation of heat-labile side chains (53).

The profound impact of extraction methods on LBP structure was systematically demonstrated by Zhou et al. (24), who compared the effects of hot water, acid, and alkali extraction, as well as sequential extraction. Using advanced characterization techniques including GPC-MALLS, AFM, and ¹H NMR, their study confirmed that extraction conditions are a key factor in shaping polysaccharide structure. This seminal work established that the extraction process is not merely a means of “obtaining” polysaccharides, but actively “sculpts” their final architecture. Their results revealed that HWE typically yields pectin-like polysaccharides with a backbone of α-1,4-D-galacturonic acid and side chains composed of rhamnose, arabinose, and galactose. ​In contrast, alkaline extraction (e.g., with 0.6% NaOH) better preserved branched structures (e.g., rhamnogalacturonan I domains), resulting in higher molecular weight and protein content, albeit with potential cleavage of acid-sensitive glycosidic bonds. Acid extraction (e.g., with 0.4% HCl), however, often led to degradation of side chains (especially arabinan), yielding polysaccharides with lower molecular weight, simpler structures dominated by more acid-resistant homogalacturonan regions.

Moving beyond conventional HWE, other methods exert distinct structural effects. Ultrasound-assisted extraction (UAE) leverages cavitation effects to efficiently break down cell walls. However, the intense mechanical shear forces can simultaneously fragment polysaccharide chains, thereby reducing the average molecular weight of the extracted LBP. Furthermore, techniques such as enzymatic extraction, microwave-assisted, subcritical water, and freeze-thaw methods have also been applied. These methods enhance extraction efficiency through unique mechanisms (e.g., specific enzymatic cleavage, rapid heating) but inevitably modify the primary structure and higher-order conformations of the resulting polysaccharides (54–56). For example, Quan et al. (57) obtained three novel *Lycium barbarum* leaf polysaccharide fractions with different molecular weights by optimizing a combined ultrasound-microwave extraction process, highlighting the ability of modern techniques to tailor polysaccharide properties.

Microbial fermentation is an efficient method for polysaccharide extraction that utilizes extracellular enzymes secreted by specific microorganisms to degrade plant cell walls, thereby facilitating the release of intracellular polysaccharides. According to a systematic review by Wang et al. (58), probiotic fermentation generally enhances the extraction yield of plant polysaccharides. For instance, lactic acid bacteria fermentation increased the yield of asparagus polysaccharides from 6.23 g/L to 10.34 g/L, while yeast fermentation raised the extraction rate of indigo fruit polysaccharides from 5.57% to 5.84%. Similarly, Yang et al. (59) reported that combined fermentation with lactic acid bacteria and yeast increased the carbohydrate content of yam polysaccharides (CYP-LS) from 71.03% to 78.49%, suggesting improved purity and yield. Further supporting these findings, Wang et al. (60) demonstrated that yeast-fermented yam polysaccharides (CYP-SC) exhibited an increase in carbohydrate content from 71.03% to 84.86%, accompanied by a reduction in protein and phenolic compounds. These results collectively highlight the efficacy of microbial fermentation as a viable strategy for optimizing polysaccharide extraction, particularly for medicinal plants such as yam and *Lycium barbarum*.

Fermentation enhances not only the extraction yield but also the functional properties of polysaccharides by modifying their structural characteristics. In a comparative study of hot water extraction versus yeast fermentation for LBP, Wang et al. (61) demonstrated that fermentation significantly reduced the molecular weight from 5.304 × 10^6^ g/mol to 2.231 × 10^4^ g/mol and improved molecular uniformity, as indicated by a decrease in polydispersity from 1.732 to 1.271. These structural changes led to increased exposure of hydroxyl groups, which contributed to enhanced antioxidant activity. Further mechanistic investigation revealed that fermented LBP extended lifespan and improved skin permeability in *Caenorhabditis elegans* by upregulating the expression of the daf-16 and sod-3 genes, underscoring its potential for anti-aging applications.

### 3.2 The Influence of origin on the structure of *Lycium barbarum* polysaccharides

The geographic origin is a key factor influencing plant secondary metabolites, and substantial evidence indicates the existence of recognizable structural differences in LBP from different growing regions. For instance, Zhao et al. (19) successfully distinguished LBPs from Ningxia, Xinjiang, Gansu, and Qinghai in China using acid hydrolysis fingerprinting combined with chemometric analysis, suggesting systematic differences in their fine chemical structures or compositional ratios sufficient for origin traceability. Furthermore, another study found significant variations in the content of cell wall polysaccharides and the expression levels of related genes in *Lycium barbarum* fruits from different origins (62). Despite these observable differences, research conclusions regarding the impact of origin on the core structure of LBP are not entirely consistent. Several studies have pointed out that although LBPs from different origins vary in yield and bioactivity, their fundamental molecular structures exhibit a high degree of similarity (63–65). Another study suggested (66) that the species has a far greater influence on the LBP structure than the geographic origin, implying that the basic structural skeleton of LBP is likely strictly regulated by genetic traits and is not easily fundamentally altered by environmental factors.

In summary, a comprehensive analysis of these findings suggests that while the macro-characteristics of LBP, such as its backbone structure, may be relatively conserved within a species, finer structural features—including molecular weight distribution, branching degree, side chain composition, terminal residues, and the relative proportions of different polysaccharide components—are likely subject to adaptive changes influenced by climatic and soil conditions of the origin.

## 4 Structure-activity relationship of the immunomodulatory activity

As a key “medicine food homology” resource, *Lycium barbarum* has been traditionally employed in Chinese medicine to tonify the liver and kidneys, boost essence and brighten the eyes, and strengthen immune function (67). Modern pharmacological studies attribute its diverse bioactivities to a key class of active compounds known as LBP (68). Research has demonstrated that LBP possesses a broad spectrum of pharmacological activities, including antioxidant, anti-aging, neuroprotective, anti-tumor, and notably, immunomodulatory effects (69). It is particularly noteworthy that LBP exhibits a unique bidirectional immunomodulatory capacity, making it a hotspot for research into functional foods and novel immunotherapies. However, the immunomodulatory activity of LBP is highly dependent on its chemical structure. Native LBP comprises structurally complex glycoconjugates (e.g., glycoproteins or proteoglycans), which often feature a broad molecular weight distribution, diverse monosaccharide composition, and intricate glycan structures. Compelling evidence shows that minor differences in structural parameters, including molecular weight, monosaccharide composition and molar ratios, types of glycosidic linkages, and three-dimensional conformation, are key determinants of the strength and target selectivity of LBP’s immunomodulatory activity (70). Consequently, a systematic investigation into the structure-activity relationship of LBP is essential, serving both as a fundamental scientific endeavor to decipher its immunomodulatory mechanisms and as a practical prerequisite for establishing standardized quality control, refining manufacturing protocols, and ultimately informing the design of effective, targeted immunotherapies.

### 4.1 Molecular weight

Immunomodulatory activity is not an inherent property of polysaccharides but is fundamentally governed by their molecular weight, which acts as a critical structural determinant (71, 72). Functioning as a biological switch, molecular weight finely regulates the intensity and nature of the immune response through several distinct mechanisms. It primarily influences the spatial conformation and solubility of polysaccharides, thereby modulating their recognition by pattern recognition receptors on immune cells. An optimal molecular weight range exists for maximal activity, with deviations leading to diminished effects. Additionally, molecular weight affects physical properties such as viscosity, which in turn governs the efficiency of *in vivo* transport and absorption. Therefore, precise control of molecular weight is essential for developing effective and predictable polysaccharide-based immunomodulators. Molecular weight is a primary factor influencing the immunomodulatory activity of LBP. Substantial evidence indicates that LBP fractions of different molecular weight ranges exhibit significant differences in their immunoregulatory effects, suggesting a complex, non-linear relationship rather than a simple correlation. For instance, Feng et al. (16) fractionated crude LBP into high molecular weight (HMW, >10 kDa) and low molecular weight (LMW, <10 kDa) components using ultrafiltration. Their results demonstrated that the HMW fraction was significantly more effective than the LMW fraction in enhancing the viability and proliferation of immune cells like RAW 264.7 macrophages, implying that HMW polysaccharides are the primary contributors to LBP’s direct immunostimulatory effects. Furthermore, the HMW LBP was more potent in promoting the production of key immune-related cytokines, including nitric oxide (NO), tumor necrosis factor-alpha (TNF-α), and interleukin-6 (IL-6), highlighting its superior capacity to activate innate immunity. However, the optimal immunomodulatory activity appears to reside within a specific medium molecular weight range. Contrary to the findings emphasizing HMW components, Guo et al. (73) reported that LBPs with a medium molecular weight (1×10^5^ ~ 3×10^5^ Da) possessed the strongest immunostimulatory activity, while activity diminished significantly when the molecular weight was below 10 kDa. This notion of an optimal mid-range is further supported by Zeng et al. (45), who observed that polysaccharide fractions with moderate molecular weights (7.481 kDa and 46.239 kDa) effectively enhanced immune responses in zebrafish and mouse models, whereas a much smaller oligosaccharide (1.912 kDa) showed weaker activity. The underlying mechanism for this phenomenon may be that a moderate molecular size favors effective multivalent binding with pattern recognition receptors on immune cells, thereby optimally triggering downstream signaling pathways.

In summary, molecular weight is a critical determinant of LBP’s bioactivity, and its effect is characterized by a complex dependency where optimal activity is often associated with a specific medium molecular weight range.

### 4.2 Monosaccharide composition

The monosaccharide composition and molar ratios are critical factors determining the immunomodulatory activity of LBP, which are typical heteropolysaccharides (74, 75). LBP is primarily composed of arabinose (Ara), galactose (Gal), rhamnose (Rha), xylose (Xyl), mannose (Man), and glucose (Glu), with Ara and Gal often being the most abundant neutral sugars. Furthermore, the presence of uronic acids (e.g., galacturonic acid and glucuronic acid) confers acidic properties to many LBP fractions (76). It is important to note that the relative ratios of these monosaccharides vary significantly depending on the source, batch, and extraction methods, leading to substantial heterogeneity in bioactivity and posing challenges for standardization and structure-activity relationship (SAR) studies. Despite this variability, a high content of arabinose and galactose is frequently observed in immunopotent LBP fractions, suggesting these sugars form an important structural basis for its activity. This notion is supported by several lines of evidence (75, 77, 78). For instance, Liang et al. (79) isolated and identified a more structurally clear Arabinogalactan-rich LFP-80-W1 from *Lycium barbarum*, which exhibited strong immune activity. Zhang et al. (80) demonstrated that LBP activates macrophages via TLR4, a process dependent on its Ara and Gal residues, indicating these sugars may serve as key recognition motifs for pattern recognition receptors (PRRs). In a detailed compositional analysis, Gong et al. (74) purified three LBP subfractions (LBGP-I-1, I-2, I-3) and found their monosaccharide profiles differed markedly; notably, LBGP-I-3, with the highest combined content of Ara (48.15%) and Gal (44.44%) and no glucose, exhibited the strongest activity in enhancing NO production, phagocytosis, and acid phosphatase activity in macrophages. Collectively, these findings underscore the significance of a high arabinose and galactose content, often associated with an arabinogalactan-like structure, as a key determinant of LBP’s immunostimulatory properties.

### 4.3 Glycosidic linkages and branching structure

The bioactivity of polysaccharides is highly dependent on their fine chemical structures, particularly the types of glycosidic linkages (α/β configuration, linkage positions), which are critical determinants of their three-dimensional conformation and biological functions (81). Recent advances in separation and analytical techniques have enabled more precise investigations into the function of specific structural motifs. An important study indicated that the presence of α-1,4-D-galacturonic acid and α-1,5-arabinofuranosyl linkages in LBP significantly influences its ability to activate macrophages, suggesting that LBP’s immunomodulatory activity may result from the synergistic effects of multiple glycosidic linkage motifs rather than a single active structure (82). To further elucidate the structure-activity relationship of LBP glycans, Peng et al. (83) systematically investigated an immunologically active arabinogalactan (LRGP3). They employed a combination of enzymatic and chemical methods, including exo-α-L-arabinofuranosidase digestion, acetolysis, and mild acid hydrolysis, to obtain specific structural fragments. Their findings revealed that the enzyme-resistant core (LRGP3-AF), obtained after removing arabinosyl side chains, exhibited significantly enhanced complement-fixing activity, indicating the importance of the internal galactan core. Furthermore, a product composed solely of galactose (LRGP3-T) showed lower activity than LRGP3-AF, suggesting that both 1,3-linked galactosyl residues and 1,6-linked galactan side chains are involved in complement activation. Subsequent partial acetolysis of LRGP3 yielded a linear 1,3-linked galactan (LRGP3-B). The reduced complement-activating activity of LRGP3-B compared to the native LRGP3 indicated that oligosaccharide side chains are crucial for full activity expression. However, the isolated oligosaccharide side chain fraction (Oligo-S) itself showed weak activity, leading to the conclusion that the arabinosyl side chains must be attached to the active 1,3-linked galactan backbone to fully express the complement-activating activity.

### 4.4 Impact of sulfate modification

Sulfation is the most extensively studied and demonstrably effective chemical modification strategy for LBP. Indeed, multiple studies have shown that introducing sulfate groups onto the sugar rings of LBP can significantly enhance its immunomodulatory activity (84). In a representative study, Wang et al. (85) performed sulfation modification on LBP using the classic chlorosulfonic acid-pyridine method. Structural characterization confirmed the successful introduction of sulfate groups, as evidenced by a decrease in molecular weight and the appearance of new characteristic absorption peaks in the infrared spectrum corresponding to S=O and C-O-S stretching vibrations. Functionally, the LBP with high molecular weight and moderate sulfate content (sLBPS) exhibited markedly enhanced immunomodulatory activity compared to its native counterpart. Specifically, sLBPS was more potent in promoting the proliferation of mouse splenic lymphocytes, increasing the ratios of CD4^+^ and CD8^+^ T cell subsets, and stimulating the production of key cytokines such as IL-2, IL-6, IFN-γ, and TNF-α. These findings strongly suggest that the increased negative charge density and the specific introduction of sulfate groups onto the polysaccharide backbone are critical structural factors augmenting its immunostimulatory effects.

To conclude, sulfation is a highly efficient strategy for augmenting the immunoregulatory activity of LBP. By introducing sulfate groups onto the molecular chains, this modification improves the physicochemical properties of LBP and, more importantly, potently enhances its ability to stimulate immune cell proliferation, regulate the balance of T-cell subsets, and induce the secretion of critical cytokines. Research further reveals that the immunomodulatory activity of sulfated LBP (sLBPS) is strongly correlated with structural parameters like molecular weight and degree of substitution, providing a robust theoretical basis for the rational design of structures to fine-tune its immunoregulatory effects and highlighting significant potential for future applications.

### 4.5 Spatial conformation

The conformational characteristics of polysaccharides are closely related to their bioactivities, which is critical for understanding their structure-activity relationship and represents an essential component of polysaccharide structural characterization (86, 87). Techniques commonly used to study polysaccharide conformation include Congo red assays, scanning electron microscopy (SEM), atomic force microscopy (AFM), and X-ray diffraction (XRD) (88). Numerous studies utilizing these techniques have revealed the conformational characteristics of LBP and their functional associations. According to Zhou et al. (24), polysaccharides obtained via alkaline extraction typically display an irregular, coiled conformation, while acid or hot water extraction favors the formation of rigid chain conformations. Zeng et al. (45) demonstrated through Congo red assays and atomic force microscopy that the high-molecular-weight pectic fractions LBPs-2 and LBPs-3 adopt a triple-helical conformation in solution, a higher-order structure that is critically linked to their immunoregulatory effects. Structural characterization by Fakhfakh et al. (28) using XRD and SEM indicated that LBP is a semi-crystalline polymer possessing an irregular, layered surface morphology. Further work by Al-Wraikat et al. (70) showed that while moderate degradation can unveil additional active moieties, excessive degradation compromises the triple-helical structure, resulting in a decline in biological activity. Current systematic investigations into the conformation of LBP are still lacking; therefore, a deeper exploration of its conformational features represents a critical future direction for fully elucidating its structure-activity relationship.

## 5 Downstream signaling pathways and the achievement of specific anti-tumor immunity

Specific anti-tumor immunity is characterized by the immune system’s precise recognition and clearance of cells bearing tumor-specific antigens. This response is chiefly executed by cytotoxic T lymphocytes (CTLs), whose potent activation is absolutely reliant on co-stimulatory signals and cytokines delivered by antigen-presenting cells, notably dendritic cells (89). As a key initiating event in the adaptive immune response, LBP acts as an important “trigger” in this immunological cascade by regulating dendritic cell function.

### 5.1 Modulation of macrophage polarization

As core components of the innate immune system, macrophages possess remarkable plasticity. In response to varying signals within the microenvironment, macrophages can polarize into subsets with divergent functions: the pro-inflammatory M1-type and the anti-inflammatory/repair-oriented M2-type (90). A balanced M1/M2 polarization state is essential for immune homeostasis, and an imbalance is a critical pathological event in numerous conditions such as inflammatory diseases, autoimmunity, and tumors (91, 92). Initial research indicated that LBP regulates the secretion of M1-type markers, including NO, TNF-α, and IL-6, in RAW264.7 macrophages, hinting at its capacity to promote an M1-like phenotypic shift (93, 94). Later work substantiated that LBP goes beyond merely boosting macrophage activity to directly “driving their polarization,” signifying an evolution in the research focus from broad “immunopotentiation” to specific “polarization modulation” (16). Investigating the *in vivo* effects of LBP, Wang and colleagues (95) demonstrated in a DSS-induced colitis mouse model that oral LBP ameliorated disease pathology, an effect linked directly to the modulation of macrophage polarization. At the mechanistic level, this study revealed that LBP inhibits LPS/IFN-γ-triggered STAT1 phosphorylation, thereby curbing M1 polarization, while concurrently boosting IL-4-induced STAT6 phosphorylation to foster M2 polarization. This provided the first clear molecular depiction of LBP recalibrating macrophage phenotype by differentially regulating STAT pathways—inhibiting one while activating another. LBP also modulates macrophage function through pathways other than STAT signaling. For example, it antagonizes LPS-induced inflammation by reprogramming cellular glycolysis and facilitating the degradation of PKM2 (96). Biological signaling pathways are not isolated linear routes but rather form a complex, dynamic, and interconnected network. A body of evidence indicates that extensive and sophisticated crosstalk exists between the NF-κB and STAT6 signaling pathways, characterized predominantly by synergistic interactions and reciprocal inhibition. Shen and colleagues (97) provided evidence via co-immunoprecipitation experiments for a direct physical interaction between STAT6 and the NF-κB p50 subunit in the cellular context. This physical association underlies their functional cooperation. Investigations into the mechanism show that the STAT6-NF-κB interaction increases the mutual DNA-binding affinity of each factor for its cognate promoter elements and facilitates the cooperative recruitment of co-activators like CBP/p300. This assembly of a more potent transcription complex drives synergistic upregulation of target genes. In other contexts, however, STAT6 activation can exert an inhibitory effect on NF-κB signaling. By competitively sequestering limiting co-activator proteins like CBP/p300, activated STAT6 can indirectly repress NF-κB-driven transcription by depriving it of these essential resources (98). Additional studies indicate that STAT6 may also inhibit NF-κB activity by stabilizing IκBα, via suppression of its phosphorylation and subsequent degradation, which in turn blocks NF-κB nuclear translocation (99). In a reciprocal manner, NF-κB activation can also impinge upon STAT pathway activity (100). For instance, NF-κB is capable of inducing the expression of Suppressors of Cytokine Signaling proteins, which serve as canonical negative-feedback regulators of STAT signaling (101). Consequently, this complex interplay of crosstalk dictates that the cellular response to stimuli such as IL-4 and pro-inflammatory cues is determined by the integrated equilibrium of STAT6 and NF-κB pathway activities.

Additionally, by acting as a TLR4 agonist, LBP activates the MAPK (p38, ERK, JNK) and NF-κB signaling cascades, leading to increased production of pro-inflammatory cytokines and NO (102). Collectively, these findings demonstrate that LBP orchestrates macrophage polarization via a multi-target and multi-pathway approach, laying a solid molecular foundation for its therapeutic application in relevant diseases.

### 5.2 Activation and functional reprogramming of dendritic cells

As the “sentinels” of the immune system, dendritic cells (DCs) are pivotal in recognizing pathogens and in the initiation and modulation of adaptive immunity. Resting, immature DCs are highly proficient at capturing antigens. Following stimulation by PAMPs or DAMPs, they undergo a multifaceted “functional reprogramming,” which involves migrating to lymphoid organs, upregulating the expression of MHC and co-stimulatory molecules (like CD80, CD86, CD40), and secreting pro-inflammatory cytokines such as IL-12 and TNF-α, culminating in their maturation into cells competent for priming naïve T cells (103). Against this backdrop, Zhu and colleagues provided a systematic account of how LBP activates murine bone marrow-derived dendritic cells (BMDCs). The research demonstrated that LBP markedly enhances the surface expression of the co-stimulatory molecules CD40 and CD86, along with MHC class II, on DCs, representing critical indicators of their phenotypic maturation. At a functional level, DCs treated with LBP showed a robust increase in their capacity to stimulate allogeneic T cell proliferation (in an MLR assay) and were potent inducers of the critical Th1-polarizing cytokine IL-12. Having established LBP’s capacity to induce DC maturation, subsequent investigations focused on elucidating the underlying mechanisms. Zhu and colleagues (104) uncovered that the action of LBP is mediated through the activation of the pattern recognition receptors TLR2 and TLR4 expressed on DCs. Activation of these TLRs triggers the downstream NF-κB signaling cascade. As a pivotal transcription factor, NF-κB enters the nucleus and drives the expression of genes involved in DC maturation and inflammation, such as those encoding co-stimulatory molecules and cytokines including IL-12 and TNF-α. Importantly, the immunomodulatory influence of LBP on DCs extends beyond the TLR/NF-κB axis. The Notch signaling pathway, essential for controlling cell differentiation, proliferation, and fate decisions, is also a key player in immune cell regulation (105, 106). Research by Wang and colleagues (107) found that LBP additionally upregulates components of the Notch signaling pathway in DCs, including Notch, Jagged, Hes1, and Hes5. When Notch signaling was inhibited, the LBP-driven maturation of DCs, their secretion of IL-12, and their capacity to amplify CTL-mediated tumor cell killing were all markedly impaired. This finding identifies the Notch pathway as another key mechanism by which LBP sculpts the immunogenic profile of DCs, specifically their ability to elicit anti-tumor immune responses. In addition to the TLR and Notch pathways, other signaling mechanisms contribute to LBP’s effects. Duan and colleagues (108) demonstrated that LBP also activates the Erk1/2 pathway through TLR4. The activated Erk1/2 then inhibits the expression of the transcription factor Blimp1, which acts as a negative regulator of DC maturation. Thus, via the TLR4-Erk1/2-Blimp1 axis, LBP alleviates the brake on DC maturation, facilitating their functional activation. More recently, the investigative focus has broadened to include metabolic regulation. Moreover, Zhang and colleagues (109), employing metabolomics, observed that LBP treatment causes profound shifts in the intracellular metabolome of DCs. The resultant metabolic signature is consistent with the bioenergetic and biosynthetic requirements of DC activation, manifesting as increased glycolytic flux to rapidly produce ATP and supply carbon skeletons for anabolic processes. This work unveiled that the functional remodeling of DCs by LBP encompasses not just signaling and phenotypic alterations but also a fundamental “metabolic reprogramming.”

### 5.3 Driving effector functions of cytotoxic T lymphocytes

Cytotoxic T lymphocytes (CTLs), or CD8^+^ T cells, serve as the primary effectors of the adaptive immune response and are crucial for the clearance of virally infected and malignant cells (110, 111). The immunostimulatory effect of LBP on CTLs is mediated primarily through the activation of dendritic cells (DCs). More specifically, LBP prompts DCs to release Th1-polarizing cytokines like IL-12, a key factor for CTL differentiation, expansion, and sustained effector function (112). In addition to this DC-mediated indirect pathway, LBP also acts directly on the tumor immune microenvironment (TIME) to foster conditions conducive to CTL activity. The TIME is often characterized by an abundance of immunosuppressive elements, including regulatory T cells (Tregs) and inhibitory cytokines such as TGF-β and IL-10 (113). Research indicates that LBP inhibits the functionality of Tregs and diminishes the production of suppressive factors such as TGF-β1 and IL-10, effectively counteracting the tumor’s strategies for immune escape (114). Supporting experimental evidence demonstrates that *in vivo* administration of LBP markedly enhances the proliferation and activation of CD4^+^ helper T cells and CD8^+^ cytotoxic T cells. Additionally, other research suggests a potential role for LBP in activating natural killer (NK) cells, which would provide an additional boost to the innate arm of anti-tumor immunity (115).

### 5.4 Modulation of regulatory T cells

Regulatory T cells (Tregs) play a central role in maintaining immune homeostasis by curbing excessive immunity and preventing autoimmunity, and their dysregulation is implicated in a spectrum of pathological conditions. A fundamental pathological feature of autoimmune disorders is impaired Treg function or reduced Treg numbers, which disrupts immunological tolerance to self-antigens. LBP exhibits considerable therapeutic promise, the mechanisms of which are strongly associated with the potentiation of Treg activity. A study by Guo and colleagues (116) revealed that a *Lycium barbarum*-derived glycopeptide markedly enhanced Treg cell expansion in the experimental autoimmune encephalomyelitis (EAE) model, which mimics multiple sclerosis. This Treg expansion occurred concurrently with the inhibition of differentiation of pathogenic CD4^+^ T cell subsets, namely Th1 and Th17 cells, leading to a significant amelioration of disease symptoms. These findings provide direct evidence for the Treg-promoting effect of this *Lycium barbarum* component in an autoimmune setting. Using a murine model of primary Sjögren’s syndrome, Wang and colleagues (117) reported that low-dose LBP successfully regulated T cell differentiation. The effects were characterized by a significant reduction in the populations of effector T follicular helper (Tfh) and Th17 cells, alongside a pronounced increase in the Treg/Tfh and Treg/Th17 cell ratios. This suggests that LBP suppresses autoimmunity by rebalancing T cell subsets, thereby strengthening the relative supremacy of the regulatory Treg population. Additionally, clinical insights from Geng and colleagues (118) in rheumatoid arthritis patients showed that LBP could rectify the dysregulated CCR9+ Th17/Treg ratio, offering further support for its anti-inflammatory potential through Treg regulation. Unlike their protective function in autoimmune diseases, Tregs are typically viewed as facilitators of tumor immune evasion in the context of cancer (119). Research indicates that LBP might potentiate CD8^+^ T cell-mediated anti-tumor immunity by diminishing the Treg population within tumors (114). While direct evidence remains limited, this points to a highly context-dependent nature of LBP-mediated Treg modulation. While the modulatory impact of LBP on Tregs is increasingly recognized, the exact molecular mechanisms and signaling pathways involved are still not fully understood. Some evidence suggests that LBP can directly target key transcription factors in T cells. For instance, LBP has been shown to inhibit the activity of the transcription factor AP-1 concurrently with promoting Treg expansion (120). Since AP-1 is a master regulator of T cell activation, proliferation, and differentiation, its inhibition by LBP could represent a critical mechanism for redirecting T cell fate towards the Treg pathway.

### 5.5 Immunomodulation via the gut microbiota

Often termed the host’s “second genome,” the gut microbiota is a complex ecosystem that resides in the gastrointestinal tract and is crucial for digestion, metabolism, and, importantly, the development and regulation of the immune system (121, 122). LBP, a characteristic botanical polysaccharide, possesses a complex structure that is resistant to enzymatic breakdown in the upper GI tract, enabling its intact delivery to the colon where it serves as a substrate for selective fermentation by gut microbes. LBP mediates its immunoregulatory functions indirectly via this critical “gut-microbiota-immune” axis (123, 124). Substantial evidence indicates that LBP administration markedly remodels the composition of the intestinal microbiota. For instance, in a cyclophosphamide (CTX)-induced immunosuppressed mouse model, Ding et al. (125) found that LBP not only restored immune function (e.g., improved immune organ indices, promoted T cell subset balance) but also effectively reversed CTX-induced gut microbiota dysbiosis. Furthermore, they identified negative correlations between the abundance of specific bacterial families (e.g., *Lachnospiraceae*, *Ruminococcaceae*) and systemic cytokine levels (e.g., IL-2, IL-6, IL-1β, TNF-α, IFN-γ), strongly implicating the microbiota as a crucial mediator of LBP’s effects. Supporting this, Wang and colleagues (126) reported that LBP specifically enriched beneficial bacterial families like *Rikenellaceae*, *Prevotellaceae*, and *Bifidobacteriaceae*, and these shifts were positively associated with improved immune markers. Notably, the regulatory effect of LBP on the microbiota demonstrates a high degree of selectivity. A body of evidence (124, 127, 128) indicates LBP fosters the growth of beneficial bacteria, including: (1) lactate producers (e.g., *Bifidobacterium*, *Lactobacillus*) that acidify the milieu and modulate immunity; (2) mucin degraders like *Akkermansia muciniphila*, which enhance barrier integrity; and (3) SCFA producers such as Prevotellaceae and Rikenellaceae. Simultaneously, LBP consumption suppresses the proliferation of potential pathobionts, leading to an optimized global microbiota composition (129). Collectively, the LBP-driven systemic restructuring of the gut microbiota constitutes the foundation for its downstream biological activities.

The microbiota remodeling induced by LBP directly results in a shifted metabolic output, most notably an enhanced generation of short-chain fatty acids (SCFAs) (130). Fermentation of LBP by commensals produces multiple metabolites, with SCFAs (acetate, propionate, butyrate) being the most functionally characterized (131, 132). SCFAs act as a vital energy source for colonocytes (especially butyrate) and function as pivotal signaling molecules that mediate crosstalk between the microbiota and the host immune system (133, 134). G protein-coupled receptors GPR41 and GPR43 are established as the principal cell-surface receptors for SCFAs. This identification reveals that SCFAs function not only as metabolic fuels but also as crucial signaling molecules. Research indicates that GPR43 has a greater affinity for acetate and propionate, while GPR41 displays higher sensitivity to propionate and butyrate (135, 136). Furthermore, GPR109A is recognized as another key receptor for butyric acid (137). These receptors exhibit broad expression patterns, found on intestinal epithelial cells, adipocytes, and—of particular significance on immune cells such as neutrophils, macrophages, dendritic cells, and T cells (138). Studies indicate that SCFAs mediate anti-inflammatory actions via diverse mechanisms, which encompass modulating immune cell chemotaxis, the release of ROS, and the production and secretion of cytokines (139, 140). Specifically, SCFAs suppress the secretion of pro-inflammatory cytokines like TNF-α, IL-6, and IL-1β and concurrently enhance the generation of the anti-inflammatory cytokine IL-10. The activation of GPR43 by SCFAs leads to effective suppression of the NF-κB signaling pathway. Multiple studies indicate (141, 142) that GPR43 activation can bypass the classic Gαi/o-mediated cAMP inhibition and instead involve the recruitment of β-arrestin 2. In its role as a scaffolding protein, β-arrestin 2 impedes IκBα degradation, which results in the cytoplasmic retention of NF-κB and blocks its nuclear entry and subsequent initiation of pro-inflammatory gene transcription. In addition to receptor-dependent signaling, butyrate is also recognized as an effective inhibitor of histone deacetylases (HDACs) (143). Through HDAC inhibition, butyrate modifies chromatin architecture to influence gene expression, exemplified by its ability to upregulate the anti-inflammatory cytokine IL-10 and to drive the differentiation of regulatory T cells, a key immunosuppressive lymphocyte subset (144, 145).

Several studies (131, 146) have demonstrated a significant increase in fecal and systemic SCFA concentrations upon LBP treatment. SCFAs modulate immunity via several mechanisms: (1) Locally, by acidifying the gut lumen to suppress pathogens and, specifically butyrate, by strengthening the epithelial barrier via enhanced energy production and tight junction integrity (147, 148); (2) As signaling molecules, they activate specific receptors and pathways in gut-resident immune cells (149, 150); (3) Systemically, they enter circulation and influence immune responses in peripheral tissues (151). Thus, SCFAs form the crucial mechanistic link whereby LBP, acting through the microbiota, regulates host immunity, effectively converting microbial signals into whole-body physiological responses.

LBP’s immunomodulatory capacity also shows therapeutic potential in autoimmune disease models. For instance, Lai and colleagues systematically delineated the mechanism through which LBP ameliorates rheumatoid arthritis (RA) in a rat model by reshaping the gut microbiota. They found that LBP treatment reversed gut dysbiosis in RA rats, notably enriching beneficial genera like *Lactobacillusand Faecalibacterium*. This was associated with altered DNA methylation patterns in gut epithelial cells, leading to modulated expression of genes involved in inflammation and immunity, and a consequent significant reduction in arthritic severity (152). This work offers robust preclinical evidence supporting the exploration of LBP as a potential adjunctive treatment for RA.

## 6 Systematic assessment of the current state of clinical research

### 6.1 Analysis of core deficiencies in current LBP clinical research

Despite promising preclinical results, the translational potential and clinical utility of LBP must be definitively established via well-controlled clinical studies in humans. A systematic review of the current literature, however, uncovers a profound lack of clinical evidence for LBP, approaching a state that can be described as an “evidence vacuum.” Our systematic search of primary clinical trial registries, such as ClinicalTrials.gov (Medicine) (153), identified scarcely any trials specifically designed to assess the impact of purified LBP on immune function in human as a primary endpoint. Likewise, a search of core scientific databases like PubMed (154) (Resources) retrieved very few qualifying humans randomized controlled trials (RCTs) where immunomodulation was the primary outcome. This evidence deficit is also conspicuous in the Cochrane Library (Collaboration) (155), from which no systematic reviews or meta-analyses concerning LBP’s immunomodulatory actions were identified. The collective “silence” from these authoritative sources underscores the current stagnation in clinical research on this topic. Although large-scale, purpose-built RCTs are absent, a handful of sporadic and relevant human studies can be found in the literature. The evidential power of these studies, however, is generally weak. As an example, one randomized, double-blind, placebo-controlled trial in healthy older adults reported that 30-day consumption of a standardized goji berry juice (GoChi^®^) significantly elevated lymphocyte counts, serum IL-2 and IgG levels, and improved subjective well-being, with no adverse events reported (156). While the outcomes are positive, the study has significant limitations: (1) The intervention was a juice, not purified LBP, meaning the effects could be due to other constituents (vitamins, carotenoids) besides LBP; (2) Lack of standardization: Critical parameters of the LBP used (e.g., concentration, molecular weight) were not specified, hindering dose-response assessment and reproducibility. Additionally, other studies administered LBP but assessed immune function only as a secondary or exploratory outcome, not as the primary endpoint. For example, one RCT in type 2 diabetic patients focused primarily on the hypoglycemic and lipid-lowering effects of LBP, without a systematic evaluation of immune parameters (157). Another small RCT involving adolescents with subthreshold depression found that LBP alleviated depressive symptoms and noted alterations in the pro-inflammatory cytokine IL-17A, leading to speculation about an immune-related mechanism (158). However, the small sample size and the exploratory nature of the immune analysis preclude firm conclusions. Furthermore, an RCT for non-alcoholic fatty liver disease is registered (ChiCTR2000034740) but results are pending; its primary endpoints are also centered on hepatic and metabolic metrics, not immunity (159).

In conclusion, the progression of clinical research on LBP’s immunomodulatory properties is hampered by several key challenges: The foremost obstacle is the lack of standardized, well-characterized LBP preparations. The inherent structural complexity of LBP presents significant challenges in manufacturing high-purity batches with consistent properties. Secondly, designing robust clinical trials is challenging due to the undefined dose-response relationship, the lack of validated immune-specific clinical endpoints, and uncertainty in selecting the optimal patient population. Third, long-term safety data for high-dose, high-purity LBP formulations in humans are scarce, raising concerns about chronic use. Finally, comparative efficacy studies pitting LBP against standard immunomodulatory therapies are entirely lacking. Due to these factors, the immunomodulatory efficacy, safety profile, and optimal therapeutic protocol for LBP in humans remain elusive and clouded in ambiguity.

### 6.2 Improvement strategies

To overcome the current stagnation in LBP clinical research, a comprehensive, integrated strategy spanning from source to endpoint is required. The goal of this strategy is to establish a novel and rigorous scientific paradigm to drive its successful translation into evidence-based medicine. Crucially, the fundamental prerequisite for resolving these issues is the procurement of a standardized, “research-grade” LBP with homogeneous quality and clearly defined properties, as this forms the essential foundation for guaranteeing that all ensuing studies are comparable, reproducible, and scientifically robust.

A multidimensional standardization framework for LBP must therefore be implemented. This should start with raw material standardization, which explicitly defines the source cultivar (e.g., *Lycium barbarum* L. from Ningxia) and requires sourcing from authenticated producing regions to guarantee uniformity in the cultivation environment, soil, and climatic factors. Standard operating procedures must also be defined for the harvest timing, drying technique, and subsequent storage parameters. In addition, standardization of the manufacturing process is critical. Standard Operating Procedures (SOPs) should be developed to strictly define parameters for water extraction, including temperature, duration, number of extraction cycles, and the solid-to-solvent ratio. Subsequently, a standardized, multi-stage purification protocol must be adopted. Following aqueous extraction and ethanol precipitation, essential steps must encompass deproteinization, decolorization, and fractionation to separate and collect polysaccharide fractions based on specific molecular weight ranges or charge characteristics a stage that is vital for achieving an LBP preparation with greater structural uniformity. Standardized processes for dialysis and final lyophilization are also necessary. Furthermore, a standardized quality control (QC) regime is imperative. A complete quality specification dossier for LBP must be created, requiring every production batch to be analyzed and conform to pre-established specifications. Critical QC parameters should encompass: macroscopic indicators (appearance, solubility, loss on drying); purity specifications (total polysaccharide content > 95%; residual protein <1%; minimal nucleic acid contamination); and detailed physicochemical characterization (molecular weight distribution, monosaccharide composition). Establishing this end-to-end LBP standardization framework, from cultivation to final product, ensures that a consistent “investigational product” is used across all future clinical trials, thereby creating a robust basis for scientifically valid assessments of its safety and effectiveness.

Building upon this standardized material, Phase I clinical trials should be promptly commenced. These studies must be designed with clear, consistent key elements: a precisely defined target population aligned with the study aim (e.g., immunocompromised elderly, oncology patients), strict enrollment criteria, a fixed dose and appropriate intervention period informed by dose-escalation data, and primary endpoints that are objective, validated, and clinically significant. For instance, a trial evaluating LBP for preventing upper respiratory tract infections in older adults might use the “incidence of infection” as the primary endpoint. Beyond the primary outcome, a rich array of secondary endpoints should be incorporated to evaluate LBP’s safety, tolerability, pharmacokinetics, and dose-response relationships with biomarkers. To resolve the existing gap and disarray in immunological evaluation, we recommend prioritizing validated immunological endpoints. Specifically, we propose a standardized set of assays, including measures of cellular immunity (e.g., T-cell phenotypes), NK cell cytotoxicity, humoral immunity (serum immunoglobulins), and inflammatory status (e.g., TNF-α, IL-6, IL-10) with guidance on sampling time points and statistical considerations, and advocate for their implementation as core secondary endpoints across all clinical trials investigating LBP’s immunomodulatory effects. This standardized immunophenotyping panel will empower future LBP clinical research to: objectively quantify the *in vivo* biological impact of LBP; (2) probe the immunological mechanisms underpinning any clinical benefits; (3) discover potential predictive biomarkers to identify patient populations most likely to respond; and (4) facilitate the comparison and meta-analysis of data across different trials, thus accelerating scientific progress.

Once a safe dosing window is established, moderate-scale Phase II proof-of-concept trials should be conducted, targeting specific clinical indications characterized by high unmet medical need. We propose several promising research avenues: (1) Application as a vaccine adjuvant: Perform randomized controlled trials (RCTs) in cohorts receiving vaccinations, comparing the effects of “vaccine plus LBP” against “vaccine plus placebo.” Primary endpoints may include specific antibody titers and neutralizing antibody levels. This direction is among the most translationally promising, given the robust adjuvant activity of LBP observed in preclinical research (123, 160); (2) Evaluation in immunosenescence: Evaluate, in healthy older adults, whether long-term LBP supplementation can ameliorate markers of immunosenescence and reduce the incidence of infections, an approach with preliminary supporting evidence (156); (3) Adjunctive therapy for chronic inflammatory/autoimmune conditions: Investigate LBP’s potential as an add-on therapy for patients with mild forms of chronic inflammatory or autoimmune diseases, with the goals of rebalancing immune dysregulation and mitigating inflammation. The bidirectional immunoregulatory capacity of LBP could provide a therapeutic advantage over mere immunosuppression in these disorders (161).

Moreover, future clinical trials should abandon a single-marker evaluation model in favor of integrating multi-omics approaches. Analyzing patient samples collected before and after intervention using genomics, transcriptomics, proteomics, metabolomics, and gut metagenomic sequencing allows for the in-human validation of preclinical pathways, the discovery of molecular signatures predictive of therapeutic response, and the establishment of a foundation for future personalized immunomodulation strategies.

## 7 Challenges and future research directions

Research into the structure-activity relationship (SAR) of LBP has made substantial progress, yet its translation into clinical applications continues to face several key obstacles. These limitations, nevertheless, help define clear priorities for future studies.

A primary challenge is the inherent structural heterogeneity of natural LBP, which complicates the acquisition of uniform compounds and obscures precise SAR analysis. Addressing this requires a multi-dimensional strategy. First, advanced NMR techniques are needed to resolve atomic-level details such as monosaccharide sequences, linkage sites, and three-dimensional folding. Second, integrated mass spectrometry can determine molecular weight distributions, deconvolute branched sequences, and distinguish conformers. Third, computational modeling combined with experimental data is crucial; molecular dynamics simulations calibrated by NMR and Small Angle X-ray Scattering (SAXS) can capture dynamic conformations, while docking studies help predict bioactive structures. Finally, macro-scale characterization via AFM, Cryo-EM, and SAXS/WAXS reveals aggregation states and solution behavior. This integrated approach will elucidate both static and dynamic structures, laying a solid foundation for precise SAR.

Another major gap is the uncertain identity of the key immunomodulatory structural motif. Bridging this gap necessitates a shift to synthetic strategies using synthetic biology and chemoenzymatic methods. Future efforts should focus on: 1) Functional genomics using CRISPR/Cas9 and isotope tracing to identify key glycosyltransferases (GTs) involved in LBP biosynthesis; (2) Protein engineering of GTs, guided by AI and modular synthetic biology, to produce optimized enzyme cascades; (3) High-throughput structure-activity screening building defined oligosaccharide libraries and testing them via rapid immune cell assays within a “Design-Build-Test-Learn” cycle. This systematic path will definitively link discrete oligosaccharide structures to immune activity, ultimately pinpointing the minimal active motif.

The absence of standardized, pharmacologically suitable LBP preparations also poses a serious bottleneck for clinical trials. There is a clear need to develop standardized bioactive fractions as an immediate and practical objective. Defining critical quality attributes, such as specific molecular weight ranges and monosaccharide composition is crucial to guarantee consistency between batches, reproducible efficacy, and reliable clinical outcomes.

Moreover, the pharmacokinetics of LBP *in vivo* and its complex relationship with the gut microbiota remain poorly explored. Elucidating the mechanisms involving the gut microbiota is essential. Future work should apply metagenomics and gnotobiotic models to move past correlations and establish causality, clarifying how specific LBP structures are processed by gut bacteria to generate immunomodulatory signals.

Ultimately, applying SAR knowledge should progress from general immune enhancement to precise targeting. This opens the way for exploring LBP in targeted drug delivery and immunotherapy. A refined SAR will facilitate designing LBP-based targeted delivery systems or “designer” immunomodulators with customized functions for use in vaccines or autoimmune diseases.

In summary, integrating LBP into modern therapeutics requires a transition from descriptive research to targeted approaches that tackle these central challenges. By systematically connecting each challenge with a specific research direction—using structural biology to address heterogeneity, synthetic chemistry to identify the active core, pharmaceutical science to ensure quality, and microbiome studies to decipher *in vivo* mechanisms traditional knowledge can be transformed into a new generation of precision polysaccharide-based immunotherapies.

## 8 Conclusion

In summary, *Lycium barbarum* polysaccharides (LBP) represent a class of promising natural immunomodulators whose biological activity is intrinsically tied to their intricate chemical architecture. Critical structural features, including molecular weight, monosaccharide profile, and glycosidic linkage patterns orchestrate the nature and potency of LBP’s interplay with the host immune system. Current research has begun to establish correlations between LBP’s structural features and effects such as immune cell activation and cytokine secretion, and has revealed that its immunomodulatory actions are likely mediated through receptors like TLR and subsequent signaling pathways such as NF-κB and MAPK. However, translating LBP from a traditional remedy into a modern, precision immunotherapeutic requires a multidisciplinary approach to overcome challenges in structural characterization, systematic bioactivity assessment, and mechanistic delineation. A comprehensive understanding of LBP’s SAR will provide a robust foundation for developing the next generation of effective, safe, and quality-controlled polysaccharide-based immunotherapies, maximizing their potential impact in preventive medicine and health promotion.
